# Differences in Management of Older Women Influence Breast Cancer Survival: Results from a Population-Based Database in Sweden

**DOI:** 10.1371/journal.pmed.0030025

**Published:** 2006-01-17

**Authors:** Sonja Eaker, Paul W Dickman, Leif Bergkvist, Lars Holmberg, The Uppsala/Örebro Breast Cancer Group

**Affiliations:** **1**Department of Surgery, University Hospital of Uppsala and Regional Oncologic Centre, Uppsala, Sweden,; **2**Department of Medical Epidemiology and Biostatistics, Karolinska Institutet, Stockholm, Sweden,; **3**Department of Surgery, Centre for Clinical Research, Central Hospital of Västerås, Västerås, Sweden; McGill UniversityCanada

## Abstract

**Background:**

Several reports have shown that less aggressive patterns of diagnostic activity and care are provided to elderly breast carcinoma patients. We sought to investigate whether differences in the management of older women with breast cancer are associated with survival.

**Methods and Findings:**

In an observational study using a population-based clinical breast cancer register of one health-care region in Sweden, we identified 9,059 women aged 50–84 y diagnosed with primary breast cancer between 1992 and 2002. The 5-y relative survival ratio was estimated for patients classified by age group, diagnostic activity, tumor characteristics, and treatment. The 5-y relative survival for breast cancer patients was lower (up to 13%) in women 70–84 y of age compared to women aged 50–69 y, and the difference was most pronounced in stage IIB–III and in the unstaged. Significant differences in disease management were found, as older women had larger tumors, had fewer nodes examined, and did not receive treatment by radiotherapy or by chemotherapy as often as the younger women. Adjustment for diagnostic activity, tumor characteristics, and treatment diminished the relative excess mortality in stages III and in the unstaged, whereas the excess mortality was only marginally affected in stage IIB.

**Conclusions:**

Less diagnostic activity, less aggressive treatment, and later diagnosis in older women are associated with poorer survival. The large differences in treatment of older women are difficult to explain by co-morbidity alone.

## Abstract 2

A study from Sweden shows that a lower 5-year relative survival for older women with breast cancer was associated with less diagnostic activity, less aggressive treatment, and later diagnosis.

## Introduction

Age should not be a determinant for quality of care in breast cancer. In Sweden, as in many other countries, the population is ageing with a rapidly increasing number of elderly women diagnosed with breast cancer as a consequence. Today about 30% of all breast cancer patients in Sweden are 70 y or older at diagnosis [ 1]. Despite this, there have not hitherto existed any well-established clinical guidelines for the management and treatment of breast cancer in older women. One major reason is that only a few trials have included, or have been designed for, women over 70 y [ 2, 3]. Therefore, there are little empirical data to support rational clinical guidelines, which leads to uncertainty about management of elderly and, possibly, contributes to age-biased treatment decisions. Several reports have shown that less aggressive patterns of diagnostic activity and care are provided to elderly breast carcinoma patients [ 3– 5], but it is not clear what these differences mean for survival.

The aim of our study was to investigate whether the differences in management and diagnosis of breast cancer in older women have an effect on the survival. We based our analysis on a population-based clinical database including information on diagnostic activity, tumor characteristics, and treatment.

## Methods

### Setting

We used a population-based regional breast cancer register for the Uppsala/Örebro health-care region. The register was established in 1992, at the same time as formal written clinical guidelines were issued for the region with the aim to ensure that all women with breast cancer had the same opportunity to receive high-quality care. The region comprises seven counties in Sweden with a total population of about 1.9 million. The register includes individual information about vital status, age, and date of diagnosis, detection mode, tumor-stage, biological tumor characteristics, and primary surgical and oncological treatment. The register includes 97% of all breast cancer patients diagnosed within the region as validated against the mandatory reporting to the Swedish Cancer Registry. The main treatment variables and the staging information have been validated in the register and over 95% agreement was found.

Within the Uppsala/Örebro health-care region, all women aged 50–69 y are regularly invited to mammography screening for a low out-of pocket fee. Additional age groups are also invited to screening in some counties, i.e., the age group 40–49 y in five counties, the age group 45–49 y in one county, the age 70 y in one county, and the age group 70–74 y in three counties.

### Participants

Participants were all women with primary breast cancer reported to the Uppsala/Örebro regional breast cancer register from January 1992 to December 2002 (12,163 women), with follow-up until December 2003. We excluded 2,169 (17.8%) women who were under 50 y of age and 867 (7.1%) who were older than 84 y. We chose women 50–69 y as a reference group for the elderly, since women 50–69 y were uniformly offered mammography screening and had well-defined treatment recommendations [ 6]. We excluded women over 84 y, since co-morbidity was deemed to play a dominant role in the choice of treatment for the oldest. From the 9,127 (100%) women aged 50 to 84 y we excluded 68 (0.8%) women for whom we had less than 1 mo of follow-up. This left us with 9,059 (99.3%) eligible women. In Sweden, treatment recommendations for women over 70 y have been diffuse and are often left to the judgment of the treating physician.

### Ethics

The data used in the study are based on quality assurance registers legally required for quality assurance of Swedish health care. The registers are inspected by the Swedish Data Inspection Board.

### Statistical Methods

We compared the 5-y relative survival for primary breast cancer patients in different age groups (50–69, 70–74, 75–79, and 80–84 y). The relative survival ratio (RSR) is the ratio of the observed survival in the population of interest to the survival that would have been expected had the patients experienced only the age- and period-specific mortality of the general population from which they were drawn [ 7]. The general population in this study is all females in the Uppsala/Örebro health-care region. Relative survival is the survival analog of excess mortality. To study differences in survival between ages while adjusting for the confounding factors available in the dataset, i.e., stage (International Union Against Cancer [UICC] stages I, II, III–IV, and not definable due to unknown lymph nodal involvement [or tumor size]), and calendar period of diagnosis (1992–1993, 1994–1995, 1996–1997, 1998–1999, and 2000–2002), we modeled excess mortality (relative excess rate [RER] of death) using Poisson regression [ 8].

We thereafter stratified the women according to breast cancer stage to study whether the differences in survival between ages were consistent across levels of this variable.

To investigate possible differences in management and treatment of younger versus older patients, we used the Fisher's exact test, which tests the independence between age (dichotomized into 50–69 y versus 70–84 y) and the diagnostic and treatment variables. The *diagnostic* variables were proliferation status (high, low, and unknown/missing), estrogen receptor status (positive, negative, or unknown), number of lymph nodes examined (1–9, 10+, no axillary dissection, or unknown), lymph nodal involvement (positive, negative, or unknown), and tumor size (≤20 mm, 21–50, ≥51, or unknown). The proliferation is measured by s-phase and Ki67, or if data on these are missing, by Elston grade. We also examined *treatment,* classified as primary surgical treatment (mastectomy, breast-conserving, none, or unknown), oncological treatment (radiotherapy, chemotherapy, or hormonal [tamoxifen]), and type of treating clinic (main hospital in the county or local hospital). For women with advanced disease, we also examined whether they had received any neoadjuvant treatment.

We also constructed a low treatment activity index for women diagnosed with stage I–III breast cancer and for whom stage was not definable. The low treatment activity index was constructed from the evidence-based clinical practice guidelines for breast cancer [ 6]. Since we had no possibility to control for eventual co-morbidity, general health, or the women's own preferences, we used the same low treatment activity index on all women. Low treatment activity for women with stage I–II breast cancer was defined as (1) no surgery at all, (2) breast conserving (BCS) without adjuvant treatment by radiation, (3) no establishment of estrogen receptor status (except for women with negative lymph node involvement [N0] and tumor size <10 mm), (4) no adjuvant treatment by tamoxifen and/or chemotherapy in women with positive lymph nodal involvement (N1) and estrogen receptor–positive breast cancer (ER+), (5) no adjuvant treatment by tamoxifen and/or chemotherapy in women with tumor size more than 20 mm and ER+, or (6) no adjuvant treatment by chemotherapy patients with N1 and estrogen receptor–negative breast cancer (ER−). For women with a locally advanced disease, low treatment activity was defined as if (7) they had not received any neoadjuvant treatment by chemotherapy, hormones, or radiation. For women whose breast cancer stage is not definable (due to missing information on lymph nodal involvement or on tumor size), points 1–3 explained above were used as an indication of low treatment activity. We also made a separate measure of unjustified over-treatment when women with ER− breast cancer had received tamoxifen.

Lastly, we studied differences in survival between the ages while adjusting for the potential determinants (categorized as explained above) by modeling the excess mortality (RER) using Poisson regression [ 8]. To assess the effect of the different variables, we made separate models for tumor characteristics, diagnostic activity, and treatment. We also sought to determine if there was any interaction between age and type of treatment on excess mortality within stage IIB, stage III, and the unstaged.

We used the analysis of variance to explore the variation of tumor size in different age groups ( Table 1).

**Table 1:**
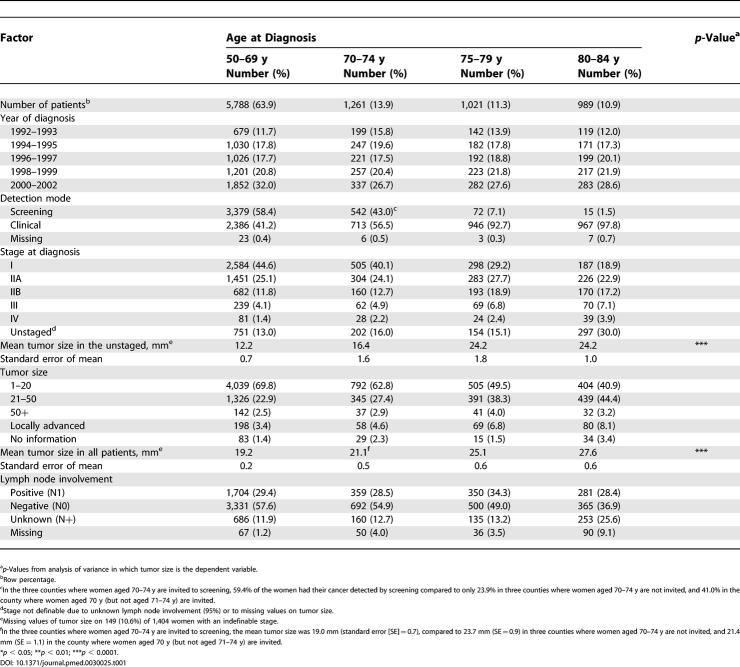
Distribution of Year of Diagnosis, Stage, and Detection Mode by Age at Diagnosis

## Results

There were large differences regarding how the breast cancer was detected, i.e., the older the woman, the lower the probability that her cancer was detected by mammography screening ( Table 1). Older women also showed a more advanced disease with larger tumors and were also more often unstaged, mainly due to missing information on lymph node involvement. The proportion of cancer detected by screening and the mean tumor size was the same for women aged 70–74 y who had been invited to screening (in three of the seven counties) as in women aged 50–69 y (see footnote to Table 1).

The unadjusted 5-y relative survival for breast cancer was significantly lower (by up to 13 percentage units) among women aged 75–79, and 80–84 y compared to that for women aged 50–69 y ( Table 2). When we adjusted for year and stage, the difference decreased somewhat ( Table 2), mainly due to the uneven distribution of stages at diagnosis (see Table 1).

**Table 2:**
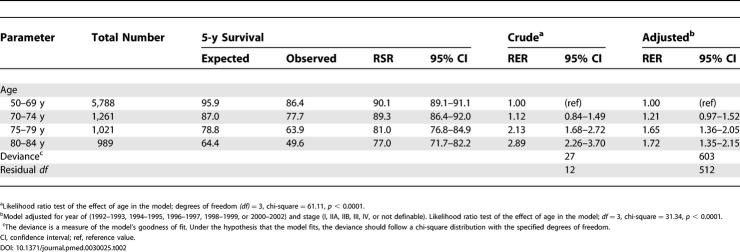
Cumulative 5-y RSR of Female Breast Cancer Patients Aged 50–84 y, Diagnosed between 1992 and 2002 (9,059 Women), and the Estimated RER and 95% Confidence Intervals

### Proportional Differences by Age in Diagnostic Activity and Treatment

Diagnostic activity and treatment differences were observed between women aged 50–69 y and 70–84 y in all stages.

#### Differences in stages I–IIB

Whereas no major proportional difference in proliferation status and estrogen receptor status were found between the ages in stages I–IIB, a *lower* proportion of the older women had had ten or more nodes examined. Regarding the treatment characteristics stage by stage, a *higher* proportion of the women aged 70–84 y, compared to the women aged 50–69 y, had received mastectomy. As regards the type of surgery delivered, radiotherapy was much less used in the elderly, stage by stage. Chemotherapy was also much less used in the elderly, in agreement with the treatment programs that regularly do not recommend chemotherapy over the age of 69 y. The low treatment activity index created for this study is also considerably *higher* in the older age group, looking at all the stages, although the differences are smaller in stage II. Tamoxifen was, however, more commonly used on the older women compared to the younger, and the difference seems to increase with increasing stage ( Table 3). A *lower* proportion of those 70 y and older are treated in the referral hospital, stage by stage.

**Table 3:**
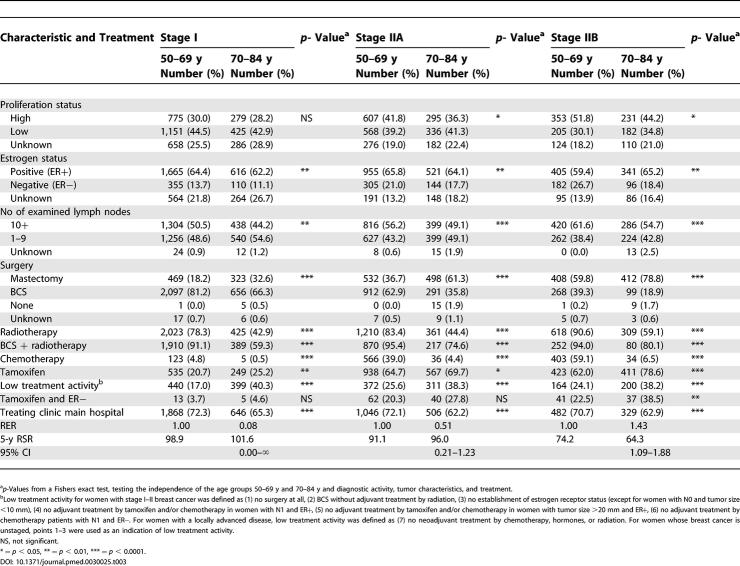
Proportions and *p-*Values of Tumor Characteristics and Treatment by Age and Stage I–II at Diagnosis, and the Cumulative 5-y RSR, RER, and 95% CI

#### Differences in stages III–IV and the unstaged

For the stages III–IV and the unstaged ( Table 4), there was an increased proportion of unknown proliferation status and estrogen receptor status in the older age groups. There were also a decreasing number of examined lymph nodes by age in those undergoing axillary dissection. Regarding the treatment characteristics for stages III–IV and the unstaged, the pattern is essentially the same as for stages I–IIB, with a lower treatment activity amongst the oldest. Although the low treatment activity index for stage III and for the unstaged is quite high for both age groups, it is higher for the older women. The older women in stages III–IV also more often did not receive any surgery at all, and, as in earlier stages, radiotherapy was much less used in the elderly as was chemotherapy. The difference in the use of tamoxifen was only present in the unstaged. A difference, as compared to the earlier stages of breast cancer, is that the differences by age group in the proportion of women treated at the main hospital of the county were not as pronounced in the higher stages. Due to the nature of the disease in the higher stages, omitting surgery is much more common in stages III and IV than in earlier stages. However, the choice not to resect the tumor is much more common in the elderly than in the age group 50–69 y.

**Table 4:**
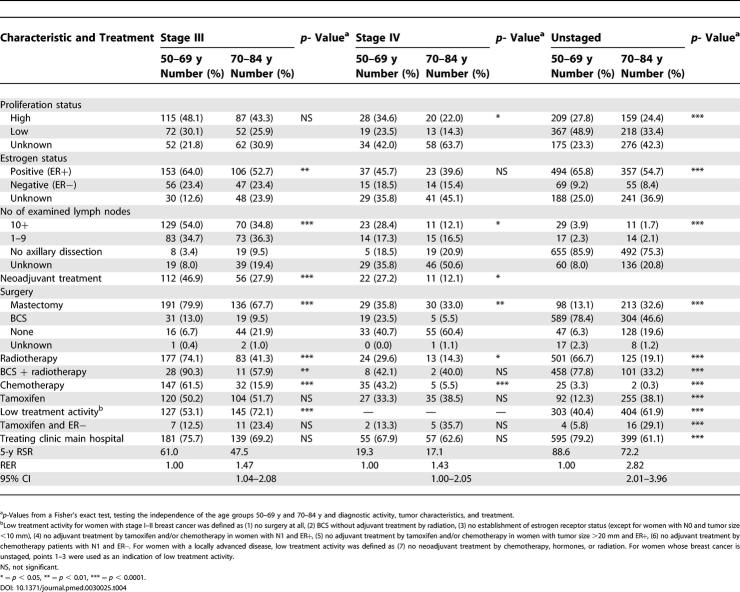
Proportions and *p-*Values of Tumor Characteristics and Treatment by Age and Stage III–IV and the Indefinable Stage, and the Cumulative 5-y RSR, RER, and 95% CI

### Adjusted Analyses

Table 5 shows the result of the statistical models in which the RER is elevated for the elderly in all models in stages IIB and higher, since these were the stages in which we found differences by age group in relative survival (see last lines of Table 3 and 4). The simpler model, which only adjusts for year of diagnosis, shows the highest RER for the older, whereas the other models tend to lower the RER as diagnostic activity, refined stage definition (by including number of involved lymph nodes and tumor size), and, finally, treatment are taken into account. In the final model, the RER for the older age group is no longer statistically significantly elevated, and any tangible difference only remains for women in stage IIB, with an RER of 1.26.

**Table 5:**
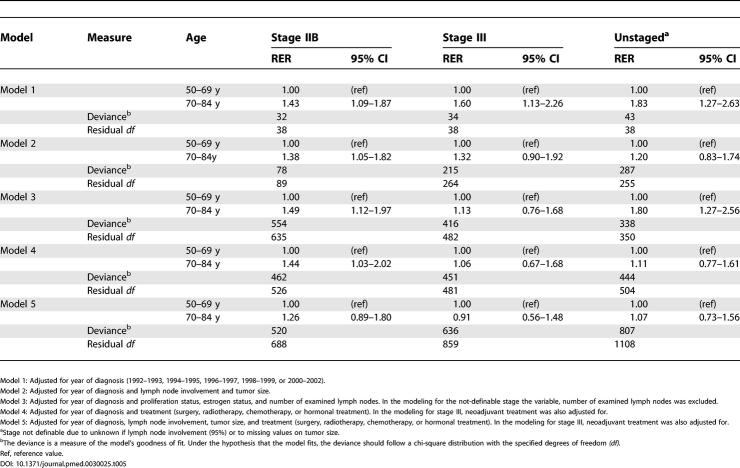
Estimated 5-y RER and 95% CI for Women in Different Age Groups, Adjusted for Year of Diagnosis, Diagnostic Activity, Tumor Characteristics, and Treatments

We found three statistically significant interactions (of 12 examined) between age and type of treatment and excess mortality, all within stage III. The RER, comparing women not receiving any surgery to those receiving surgery, was four times higher among the younger compared to the older (RER 6.4 versus 1.5, respectively), whereas the RERs comparing excess mortality among women who received radiation versus no radiation, and chemotherapy versus no chemotherapy were both lower among younger compared to the older women (RER 0.3 and 0.4 for younger women compared to 0.9 and 1.3, respectively, among the older) (data not shown).

## Discussion

We found large and clinically relevant absolute survival differences disfavoring women aged 70–84 y as compared to women aged 50–69 y. Due to the Swedish mammography screening policies, the stage distribution was more unfavorable among the older, but an adjustment for stage could only partly explain the survival differences. Looking at relative survival stage by stage, the prognosis was worse in stage IIB and higher among women over 70 y as compared to those 50–69 y. Consistently over the stages we found less diagnostic activity, less use of breast conservation, considerably less use of chemotherapy, higher “low activity index,” and more often treatment in local hospitals in older women. Adjusting for these factors in models largely explained the differences by age group, except for women in stage IIB. Our large population-based register lends further strong support to previous reports [ 4, 9, 10] that there is an age bias in management of older women.

We used a population-based database with high coverage and little misclassification of the variables used in this analysis. Another strength is that relative survival provides a measure of the excess mortality experienced by breast cancer patients, irrespective of whether excess mortality is directly or indirectly attributable to cancer [ 8]. Therefore, we could study the excess mortality adjusting for the expected survival in the background population and thereby largely compensate our comparisons for mortality due to co-morbidity. However, a drawback of the study is the lack of information on co-morbidity. Co-morbidity may have provided rational reasons for withholding diagnostic measures or specific treatments. One recent study has, however, found non-rational differences in treatment among older women even after controlling for co-morbidity [ 11]. It would also have been interesting to know whether socioeconomic status is a determinant for survival, but in essence this would not have justified an eventual age bias in management.

An important explanation for the poorer survival among the elderly was the unfavorable stage distribution due to considerably less mammography screening activity after 70 y and no screening taking place after 74 y. This explanation points to an important health policy issue as the population ages. In many Western countries today, a 70-y-old woman has a life expectancy of 12–16 y.

In stages IIB and higher, radiotherapy was less often used in older women, and this could not be explained only by the less frequent use of breast conservation in the elderly. Radiotherapy in itself, although very effective in reducing relapses also among elderly [ 2, 12] and generally well tolerated in higher age groups [ 13], will not have a major impact on mortality. However, the use of radiotherapy upon adequate indications may, in register data, be a proxy variable of the use of multidisciplinary management and compliance to clinical recommendations.

Only a very small proportion of the older women received chemotherapy. This is a consequence of the fact that the guidelines during this time period often have suggested upper age limits of 65–70 y for recommending chemotherapy. The evidence base for effectiveness of chemotherapy in the elderly is considered weaker than for younger women and only a few studies of the effect of chemotherapy have included women over 70 y [ 2]. A few studies have shown both that older women (≥65 y) have a higher treatment-related mortality than do younger women [ 14, 15], and that older women in reasonably good health derive similar benefits (in relation to disease-free survival and overall survival) from more chemotherapy as the younger patients [ 14]. We only found an effect of modification of treatment in stage III, and not in stage IIB and unstaged, strengthening these findings. Moreover, co-morbidity has been found not to be an adequate explanation for why clinicians decide to use chemotherapy [ 4], and age alone seems to be an important indicator of the likelihood to receive adjuvant chemotherapy [ 5]. The survival differences by age group found in our study and studies by others point also to the importance of further investigating effective adjuvant systemic treatments in the elderly.

Tamoxifen was widely used in older women in our study. One possible reason for the difference in use (to the disadvantage of the younger patients) is that during the first half of the 1990s, tamoxifen was considered to have a negligible effect in younger patients and was therefore not given to those who received chemotherapy. However, we also found that with higher age, an increasing proportion of women with receptor-negative tumors were treated inappropriately with tamoxifen. A possible interpretation of this finding is that clinicians felt that some treatment activity is warranted in women perceived to have a moderate or high risk of recurrence, but that, in the absence of guidelines regarding chemotherapy, the clinician took the chance that tamoxifen would provide some benefit but little harm.

In summary, elderly women were disfavored in several ways: less use of mammography screening, lower diagnostic activity, and lower treatment activity, leading to a lower relative survival. This is a very distressing finding, since around 30% of all breast cancer patients are above the age of 70 y. There is an acute need for more empirical evidence about the effectiveness and tolerability of different treatments in elderly women. Likewise, it is probable that better structured guidelines for elderly women would be a means to improve the situation.

**Patient Summary**

**Background**

Breast cancer is the most common cancer in women in much of the developed world; for example, there are 41,000 new cases in the UK per year. In 2003, 6,869 women were diagnosed with breast cancer in Sweden. Survival has improved greatly; in the past ten years in the UK, the risk of dying of the disease has fallen by one-fifth. The disease is rare in women under 30 years, but the risk of breast cancer increases with age. Although there are a number of treatments for breast cancer, previous work has suggested that certain factors may affect whether a woman gets treatment. For example, older women are less likely than younger women to be entered into trials of treatment for breast cancer, and therefore treatment guidelines are not as clear for older women. They may also be less likely to receive breast cancer screening.

**Why Was This Study Done?**

The researchers wanted to look at whether the age of women affected both their survival rate and the treatment they were likely to receive for breast cancer.

**What Did the Researchers Do and Find?**

They studied 9,059 women aged 50–84 years, diagnosed with primary breast cancer between 1992 and 2002 in one health-care region in Sweden. The researchers found that relative survival over five years of women between 70 and 84 years of age was up to 13% lower compared to women aged 50–69 years. The difference in survival was most pronounced in women who had been shown to have more advanced disease or in whom no assessment of the stage of the disease had been made. There were significant differences in disease management found; older women had larger tumors, had fewer lymph nodes examined, and did not receive treatment by radiotherapy or chemotherapy as often as the younger women.

**What Do These Findings Mean?**

In older women, the diagnosis of breast cancer was often made later than in younger women. Once diagnosed, older women were less likely to be fully investigated for their cancer, and had less aggressive treatment. It is possible that other illnesses (co-morbidities) in these women may have meant that they were less likely to survive the cancer, but this cannot be the main cause of the differences, and diagnosis in older women is associated with poorer survival. The large differences in treatment of older women are difficult to explain by co-morbidity alone. Even in a country such as Sweden with good health care, age results in great differences in the diagnosis and care of women with breast cancer, with older women faring much worse than younger women.

**Where Can I Get More Information Online?**

The US National Cancer Registry has a page with many links to information on breast cancer, including prevention and treatment:

http://www.cancer.gov/cancertopics/types/breast

Cancer Research UK, a large UK charity that funds research into breast cancer, has many pages of patient information:

http://www.cancerresearchuk.org/aboutcancer/specificcancers/breastcancer

Swedish Cancer Registry:

http://www.socialstyrelsen.se

Regional Oncologic Centre in Uppsala/Örebro Region:

http://www.roc.se

Swedish Cancer Society:

http://www.cancerfonden.com

## Abbreviations

BCS: breast-conserving surgery
CI: confidence interval
ER−: estrogen receptor–negative breast cancer
ER+: estrogen receptor–positive breast cancer
N0: negative lymph node involvement
N1: positive lymph node involvement
RER: relative excess rate
RSR: relative survival ratio.
